# Bioactive Ingredients from Dairy-Based Lactic Acid Bacterial Fermentations for Functional Food Production and Their Health Effects

**DOI:** 10.3390/nu15224754

**Published:** 2023-11-11

**Authors:** Helena Mylise Sørensen, Keith D. Rochfort, Susan Maye, George MacLeod, Christine Loscher, Dermot Brabazon, Brian Freeland

**Affiliations:** 1 School of Biotechnology, Dublin City University, D09 DX63 Dublin, Ireland; christine.loscher@dcu.ie (C.L.); brian.freeland@dcu.ie (B.F.); 2 I-Form, Advanced Manufacturing Research Centre, Dublin City University, D09 DX63 Dublin, Ireland; dermot.brabazon@dcu.ie; 3 School of Nursing, Psychotherapy and Community Health, Dublin City University, D09 DX63 Dublin, Ireland; keith.rochfort@dcu.ie; 4 Dairygold Co-Operative Society Limited, Clonmel Road, Co. Cork, P67 DD36 Mitchelstown, Ireland; smaye@dairygold.ie (S.M.); gmacleod@dairygold.ie (G.M.)

**Keywords:** lactic acid bacteria, functional food, dairy products, health benefits, vitamins, bacteriocins, bioactive peptides, bioactive compounds, fermentates, food fortification

## Abstract

Lactic acid bacteria are traditionally applied in a variety of fermented food products, and they have the ability to produce a wide range of bioactive ingredients during fermentation, including vitamins, bacteriocins, bioactive peptides, and bioactive compounds. The bioactivity and health benefits associated with these ingredients have garnered interest in applications in the functional dairy market and have relevance both as components produced in situ and as functional additives. This review provides a brief description of the regulations regarding the functional food market in the European Union, as well as an overview of some of the functional dairy products currently available in the Irish and European markets. A better understanding of the production of these ingredients excreted by lactic acid bacteria can further drive the development and innovation of the continuously growing functional food market.

## 1. Introduction

Lactic acid bacteria (LAB) are a diverse group of microorganisms that have an essential role in the food and pharmaceutical industries [1]. The bacteria in this group can generally be characterized as Gram-positive and non-spore-forming with a cocci or rod shape, and their main metabolic product is lactic acid. Organisms belonging to the genera *Lactobacillus*, *Leuconostoc*, *Pediococcus*, and *Streptococcus* are considered LAB, but it has been suggested that *Aerococcus*, *Alloiococcus*, *Carnobacterium*, *Dolosigranulum*, *Enterococcus*, *Globicatella*, *Lactococcus*, *Oenicoccus*, *Tetragenococcus*, *Vagococcus*, and *Weissella* should be included, as well [2,3].

Historically, LAB have been cultured and applied in the transformation of raw food material into fermented products such as yoghurt, cheese, bread, and a wide range of fermented meat and vegetables [4]. The fermentation process aids in the conservation of food material, enhances the texture and flavor profiles of products, and increases the health benefits that can be derived upon consumption [2]. Their traditional applications in food production have given LAB a GRAS (generally regarded as safe) status [5]. 

This review explores the diverse range of functional ingredients produced by lactic acid bacteria, including vitamins, bacteriocins, bioactive peptides, and bioactive compounds. Exopolysaccharides are another metabolite excreted by LAB, and their production, purification, and health benefits have previously been reviewed extensively and will, therefore, not be discussed in this review [6]. Additionally, lactic acid itself is a viable food ingredient produced by LAB, which has been discussed at length in several publications [7,8,9] and is, therefore, not the target of this review.

### 1.1. Food Product Regulations

The European Union (EU) has a common framework of regulations for food, which can be intricate and thorough to navigate. The objective of this framework is to guarantee that a level of food safety standards is met, and that the marketing of products does not make unsubstantiated claims and is not misleading or confusing for consumers, thereby safeguarding consumer interests [10]. The European Food Safety Authority (EFSA) provides this framework of scientific advice and risk assessments and aids in decision-making. The EFSA further evaluates the scientific claims made by companies and determines the validity and substantiation of any suggested health claims [11]. 

In order to introduce innovative products with novel food ingredients and health claims to the European market, the products need to comply with the Nutrition and Health Claims Regulations (NHCR) (Regulation EC No. 1924/2006) [12]. To receive approval for sale in the EU, the products must, therefore, adhere to the regulations set forth by the EFSA. Specifically, the following criteria must be met [13]:

- The food/constituent is defined and characterized.
- The claimed effect is based on nutrient essentiality, OR the claimed effect is defined and a beneficial physiological effect can be measured in vivo in humans.
- The food/constituent is required for bodily function, OR a cause-and-effect relationship between consumption and human health has been established.
- The quantity of the food/constituent can be consumed as part of a balanced diet to obtain the claimed effect.

Additional guidance for the determination of benefits to human health can be found in documents describing health claims related to the following [14,15]:

- Functions of the nervous system.
- Physical performance.
- Bone, joints, skin, and oral health.
- Appetite ratings, weight management, and blood glucose concentrations.
- The immune system, the gastrointestinal tract, and defense against pathogenic organisms.

This extensive framework aims to promote trust in the functional food market among consumers, but it can also be a challenge for food innovation in the food industry. The high standards of the NHCR regulations can be discouraging for producers of functional products, with “wording of claims” and “missing transparency” being reported by the industry as the major challenges [10]. In addition to this common framework, European member states can also be subjected to regional restrictions [16].

Health claims stating that microorganisms act as probiotics have not yet been authorized by the European Commission; however, some member states do allow for the use of the term “probiotic effect” [14].

### 1.2. Current Functional Dairy Products on the European Market

**Figure 1:**
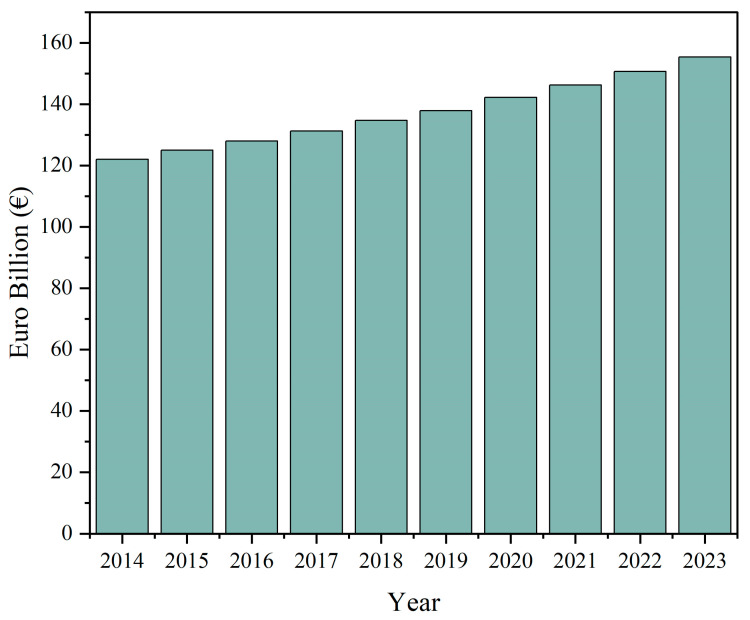
The total amount of dairy products sold in Europe in billion euros (€) from 2014 to 2023. The data include milk, yoghurt, dairy-based and soy-based desserts, cream, butter, and spreadable fats. The figure was produced with data obtained from MarketLine (https://www.marketline.com/, accessed on 6 July 2023).

The growth of the functional food market worldwide has been forecasted to continue its increase throughout the next decade, with an expected global market increase from 177.4 billion USD in 2021 to 219.5 billion USD in 2026 [6]. With an increased focus on health and nutrition after the COVID-19 pandemic, consumers are actively seeking out products with added health benefits, such as dairy products that can aid in the support of nutritional requirements or generally improved health [17]. Additionally, the dairy market is dynamic and undergoing constant development and innovation. The total amount of dairy products sold in Europe is steadily increasing according to MarketLine and will reach 155 billion EUR in 2023 (Figure 1). This, in combination with the growing interest in functionality in foods among customers, renders dairy products an optimal choice for the enhancement of fortification. 

The majority of food products on the European market with health claims deal with the addition of vitamins, minerals, protein, and/or fiber [18]. Regarding microorganisms like LAB, NHCR regulations prevent the term “probiotic” from being applied as this claim has not yet been authorized by the European Commission; however, some member states do allow the use of the term “probiotic effect” [14]. 

Table 1 provides a summary of dairy products available within the Irish and European markets, which includes kefir, yoghurt and drinking yoghurt, milk, butter, and milk powders.

A current trend among consumers is the tailoring of functional products to specific needs and population groups, in which the fortified milk range by Avonmore (Tirlán) is a good (Table 1).

Milk powders with functionality are currently mostly aimed at infant and child nutrition but are starting to see their introduction in adult nutrition, as well, with the launch of Aerabo skim milk powders from Dairygold, which is being sold on the Chinese market. Like the Avonmore range of functional milk, the Dairygold line of functional milk powders also targets its application toward different groups: Aerabo Active Vitality targets the middle-aged and senior population [19], Aerabo Active Boost is for improved immune support [20], and Aerabo Active Light is for improved nutrition and wellbeing with a lower calorie content [21].

**Table 1:** Summary of dairy products available in the Irish and European markets, which includes kefir, yoghurt and drinking yoghurt, milk, butter, and milk powders.

Product	Product Name	Functional Ingredient	Health Benefit	Company	Ref.
Kefir	Kefir Smoothie	Probiotics Calcium	Gut health	Müller, Fischach, Germany	[22]
Kefir	Kefir Yoghurt	Probiotics	Gut health	Glenillen Farm, Gurteeniher, Ireland	[23]
Kefir	Spoonable Kefir	Probiotics Calcium	Gut health	Irish Yogurts, Clonakilty, Clonakilty, Ireland	[24]
Kefir	Kefir Drink Original	Probiotics Vitamin B12 and B2 Calcium	Immune health Gut health	Biotiful Gut Health, London, The United Kingdom	[25]
Kefir	Kefir Yoghurt Original	Probiotics Vitamin B12 and B2 Calcium	Digestion Immune health Gut health	Biotiful Gut Health, London, The United Kingdom	[26]
Milk	Avonmore Super Milk	Vitamins	Bone health Nutrition	Tirlán, Ballyragget, Ireland	[27]
Milk	Avonmore Fibre Plus Milk	Vitamins Fiber	Gut health Immune health Nutrition	Tirlán, Ballyragget, Ireland	[28]
Milk	Avonmore Slimline Milk	Vitamins Iron	Nutrition	Tirlán, Ballyragget, Ireland	[29]
Milk powder	Nido©	Vitamins Minerals	Nutrition	Nestlé, Ballyragget, Ireland	[30]
Milk powder	Baby&Me© Organic	Vitamins Minerals Oligosaccharides	Nutrition	Arla, Viby, Denmark	[31]
Milk powder	Aerabo Active Vitality	Amino acids Antioxidants Glucosamine Minerals Vitamins	Bone health Nutrition	Dairygold, Mitchelstown, Ireland	[19]
Milk powder	Aerabo Active Boost	Amino acids Antioxidants Beta carotene Vitamins	Immune support Nutrition	Dairygold, Mitchelstown, Ireland	[20]
Milk powder	Aerabo Active Light	Amino acids Antioxidants Beta carotene Minerals Vitamins	Nutrition	Dairygold, Mitchelstown, Ireland	[21]
Milk drink	Goede Morgen!	Vitamins	Nutrition	Friesland Campina, Amersfoort, Netherlands	[32]
Yoghurt	Activia	Probiotics	Gut health	Danone, Paris, France	[33]
Yoghurt	Danonino	Minerals Vitamins	Nutrition	Danone, Paris, France	[34]
Yoghurt	Benecol© Yoghurt	Plant sterols	Cholesterol-lowering	Raisio Group, Raisio, Finland	[35]
Yoghurt	Natural Yoghurt	Probiotics	Gut health	Glenillen farm, Gurteeniher, Ireland	[36]
Yoghurt	Greek style natural	Probiotics	Gut health	Irish Yoghurts, Clonakilty, Clonakilty, Ireland	[37]
Yoghurt drink	Actimel	Probiotics Vitamins	Immune health	Danone, Paris, France	[38]
Yoghurt drink	Benecol© Yoghurt Drink	Plant sterols	Cholesterol-lowering	Raisio Group, Raisio, Finland	[35]

## 2. Vitamins

Vitamins are organic compounds that constitute an essential part of the human diet. Humans are for the most part unable to synthesize vitamins themselves and rely on supplementation through a balanced diet [39]. Certain LAB can synthesize some of these essential vitamins that humans are incapable of producing including folate (vitamin B9), cobalamin (vitamin B12), riboflavin (vitamin B2), and menaquinone (vitamin K2) [40,41].

**Figure 2:**
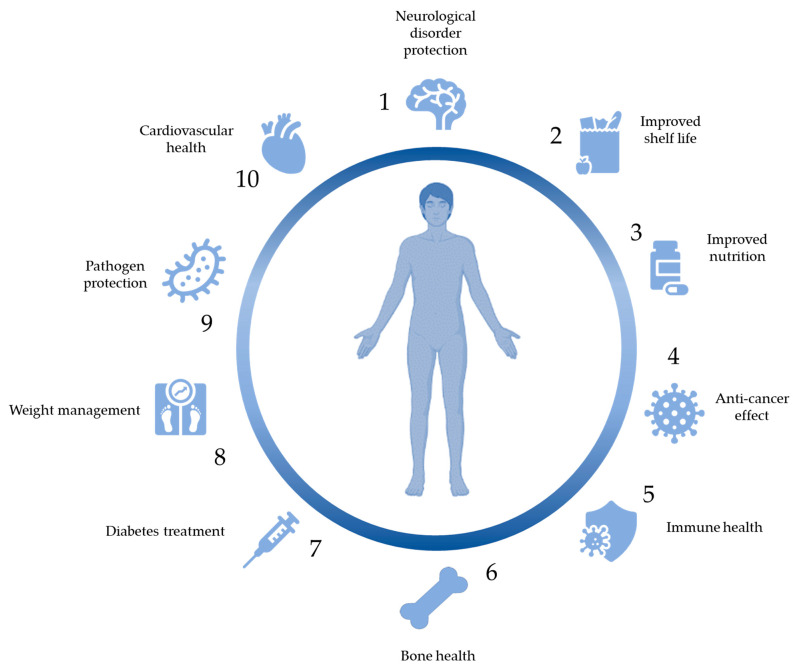
Overview of health benefits related to consumption or usage of bioactive ingredients. Specific health benefits associated with: Vitamins: 1,2, 4, 6, and 10. Bacteriocins: 2, 4, 5, and 9. Bioactive peptides: 5 and 10. Bioactive compounds: 1,2, 4, 6, and 10.

The ingestion of vitamins is widely known to be associated with a range of health benefits (Figure 2). 

### 2.1. Folate

Folate is essential for a wide range of biological functions including amino acid metabolism and DNA repair and is essential for cell division [42]. 

LAB, Bifidobacteria, and propionic acid bacteria (PAB) are all capable of producing folate while also being food-grade microorganisms (Table 2). Folate yields are, however, generally reported to be very low, even in genetically engineered strains [43,44,45]. Folate is, therefore, primarily produced synthetically as folic acid, but the human dihydrofolate reductase enzyme has an extremely low rate of folic acid conversion into bioactive vitamins and a high concentration of this synthetic form is thus needed [46]. 

#### 2.1.1. Production of Folate

Hugenschmidt et al. (2010) screened 151 LAB strains and 100 PAB strains for their ability to produce folate in a supplemented whey permeate media. Strains belonging to Lactobacillus showed the highest extracellular folate production, with *L. plantarum SM39* being the highest producer at 3.97 ng/mL [48]. This strain was subsequently used in a follow-up study examining the production of folate and cobalamin in co-culture with *Propionibacterium freudenreichii*, in a supplemented whey media. The addition of para-aminobenzoic acid (pABA) to the fermentation media increased the folate yield more than 10-fold [52]. This stimulating effect of pABA has also been observed in other studies on the strains *Streptococcus thermophilus* and *L. lactis*, where the production yield of folate was dependent on pABA in the media. This can be explained by pABA being one of the precursors of folate production. The level of pABA needed for optimal folate production is not fully described, with one study reporting that production yields did not increase with a concentration of pABA above 1 µM [53], while another reported this number to be above 100 µM [50].

Mousavi et al. (2013) tested the effect of different carbon sources (glucose, maltose, sucrose, lactose) and nitrogen sources (meat extract, yeast extract, peptone/casein, peptone/meat, (NH_4_)_2_SO_4_, and NH_4_NO_3_^2−^) to find optimal cultivation conditions for folate production of *S. thermophilus*. It was concluded that lactose and yeast extract were the optimal carbon source and nitrogen source, respectively [53]. 

Another study further supplemented media with the prebiotics mannitol and sorbitol, which increased the yield of folate due to the stimulatory effect of prebiotics on cell growth. Folate molecules are highly sensitive to oxidation, and the reducing agents sodium thioglycolate, sodium ascorbate, and cysteine hydrochloride have been added with a positive effect on the final yield of folate produced by a strain of *L. lactis* [40].

Two studies carried out fermentation of *S. thermophilus* in bioreactors and observed a high increase in folate production when pH was controlled, but where one study observed 7.3–9.3 as the optimal pH range [50], another determined pH 7 to be the optimal pH [53].

The production of folate in situ has been studied, as well. Yoghurt is an example of a product well known for its potential in being a source of folate, although the concentrations of folate are, however, highly variable [41,51]. By fermenting milk with high folate-producing strains of the known yoghurt-producing species *S. thermophilus* and *L. delbrueckii* subsp. *bulgaricus*, it was possible to increase folate content by 125% compared to commercial yoghurts [51]. Currently, commercial yoghurts can contribute 10–20% of daily levels of folate, but with both optimum strain selection and growth factors, this number has great potential for future increase [50].

#### 2.1.2. Health Benefits of Folate

Folate is an essential nutritional component that cannot be synthesized by mammalian cells, and thus it must be supplemented through the diet. The recommended daily intake of folate for adults is 400 µg/day, but varies slightly for different age groups [54]. Some population groups exhibit a higher risk of folate deficiency, which is especially prevalent in the elderly population due to lower food consumption but also in pregnant women as higher levels of folate are required to assist in neural tube development [55]. Obtaining the right amount of folate is essential as excessive intake can lead to the accumulation of folic acid in the bloodstream and mask a potential cobalamin deficiency, as symptoms are comparable [56].

Low levels of folate in the diet during pregnancy have been associated with neural tube defects, which can lead to severe consequences for the developing fetus including anencephaly (absence of major parts of the brain), spina bifida (spinal cord not formed properly), encephalocele (sac containing brain tissue protrudes through the skull), and stillbirth [57]. Accordingly, most countries recommend folate supplementation of at least 400 µg/day for pregnant women [57,58].

Folate can lower the levels of homocysteine, an amino acid that has been associated with neurodegeneration and Alzheimer’s disease [59,60]. Large studies in the UK and Sweden have indeed found an association between folate levels and cognitive function and Alzheimer’s disease [59,61]. A correlation between folate deficiency and depression and schizophrenia has also been described [62]. This was investigated in a study where patients suffering from depression and schizophrenia were supplemented with 15 mg of methyl folate per day for 6 months in addition to standard psychotropic treatment and a significant improvement of clinal and social recovery was observed [63].

The ability of folate to lower homocysteine levels can furthermore reduce the risk of cardiovascular diseases, and studies have shown an inverse relationship between folate and cardiovascular disease [64,65]. Oral administration of folate has shown the ability to reduce homocysteine levels by 25% in subjects who are not folate-deficient [66].

Folate levels might also modulate the risk of cancer, as a higher intake of folate has been associated with a lowered risk of colon cancer [67]. Balancing the intake of folate is also a necessity as it has been demonstrated how a modest supplementation of folate can reduce carcinogenesis, while an excessive amount might increase the growth of tumors [67].

### 2.2. Cobalamin

Cobalamin is an important co-factor in the metabolism of amino acids, carbohydrates, fatty acids, and nucleic acids [41]

Chemical production of cobalamin is far too expensive to be commercially viable and involves more than different 70 steps, so industrial preparation takes place through bacterial fermentation [68]. In industrial fermentations, species of *P. freudenreichii*, *Paracoccus denitrificans*, and *Bacillus megaterium* are highly used. Of these, only *P. freudenreichii* has GRAS status and is, therefore, the only one of these microorganisms that can currently be utilized directly in food production (Table 3) [44,69].

#### 2.2.1. Production of Cobalamin

The production of cobalamin from different strains of Propionibacterium in a whey-based fermentation medium has also been optimized in several studies [75,76,77,78]. The production of cobalamin by *P. shermanii* was optimized by investigating optimal levels of whey and yeast extract in the growth medium. It was determined that with a given level of whey, a certain amount of yeast extract was needed, with 10% whey solids and 1.5% yeast extract resulting in maximum yield [75].

The addition of the amino acids betaine and choline as well as diammonium hydrogen phosphate ((NH_4_)_2_HPO_4_) to a whey-based media showed an increase in cobalamin production. It was determined that the addition higher than 0.5% *w*/*v* of either of the amino acids did not increase cobalamin production further. In addition, the effect of betaine was observed to be more stimulating than choline [77,78]. This stimulating effect of betaine concurs with studies on cobalamin production of *P. denitrificans* [71,72] and is explained by betaine acting as a methyl group donor for the highly complicated cobalamin structure that contains up to eight methyl groups [71]. 

PAB are microaerophilic and produce cobalamin in high yield only when the concentration of oxygen is kept low [68]. One step in the cobalamin synthesis does, however, require oxygen, and the fermentation process producing cobalamin from *P. freudenreichii* is, therefore, often divided into two stages, as the optimal yield is dependent on both an anaerobic and aerobic phase [79]. A periodic fermentation with *P. freudenreichii* was developed with a fluctuation of oxygen supply and a cyclic operation switching between anaerobic and aerobic conditions. This operation mode resulted in a low concentration of propionic acid together with a high level of cobalamin [73]. 

One of the challenges of producing cobalamin from Propionibacterium is the accumulation of metabolites such as propionic acid and acetic acid, which harbors growth inhibitory effects [80]. In a study by Miyano et al. (2000), three different strategies were applied to circumvent this in *P. freudenreichii* fermentations: periodic changing of DO concentration between 0 and 1 ppm, a cell recycle system, and mixed culture with *R. eutropha* that is capable of assimilating propionic acid. From these three methods, the cell recycle system gave the highest cobalamin productivity, but the mixed cell system resulted in the highest cobalamin produced per unit volume of medium [74]. 

#### 2.2.2. Health Risks Associated with Cobalamin Deficiency

The recommended daily intake of cobalamin for adults is 2.4 µg/day [39], and cobalamin deficiency can occur in the elderly population, children, pregnant women, and people on a plant-based diet that excludes naturally high-cobalamin foods such as meat and dairy [81]. This deficiency is usually caused either by a lack of cobalamin in the diet or by malabsorption. It is estimated that 6% of the UK population under 60 and 20% of the population over 60 years exhibit a cobalamin deficiency. [81]. The deficiency of cobalamin typically appears with symptoms such as fatigue and anemia, but can also result in bone marrow suppression and risk of cardiomyopathy in more serious cases of deficiency [81]. Patients with a cobalamin deficiency can be ordinated oral treatments of the vitamins, while fortification of foods to make them more functional could be the solution in countries with high numbers of deficiencies in the population [82]. 

In addition to low folate levels inducing the risk of neural tube development, there is also evidence that low cobalamin levels might induce the same risk [83,84]. This area of research is still lacking in data on both need and dosage; however, it is thought that the benefits of cobalamin supplementation outweigh any potential consequences [85].

As mentioned, the levels of homocysteine appear to be related to cardiovascular disease. A study that found a reduction in homocysteine levels via supplementation with 5 mg folate also found a small but further decrease (7%) when additionally supplementing the diet with 0.4 mg/day cobalamin [86]. Most of the research on cardiovascular disease is, however, conducted on folate with or without the addition of cobalamin, making the information on the specific effect of cobalamin limited [87].

Cobalamin deficiency has also been linked to low bone mineral density, low bone mineral content, growth retardation, and increased risk of bone fraction [88,89,90]. 

### 2.3. Riboflavin

Traditionally riboflavin has been produced chemically, but the biotechnological production through strains such as *B. subtilis*, *A.gossypii*, *C. famata,* and *L. lactis* has gained more interest, with only *L. lactis* belonging to the group of LAB (Table 4) [91]. Although some strains of bacteria and yeast are considered good producers of riboflavin, the ascomycete fungi *A. gossypii* is considered the best as it can produce 40,000 times more riboflavin than it needs for its growth [92]. 

#### 2.3.1. Production of Riboflavin

*L. acidophilus* and *L. lactis* have been applied in studies examining their riboflavin production [91,93]. *L. lactis* and *L. acidophilus* were grown on both milk and whey. Whey appeared to be a more optimal growth medium for riboflavin production, and *L. acidophilus* was the optimal producer of riboflavin with a final yield of 2.93 mg/L [93]. Although this yield is significantly lower than those that are possible to obtain with *A. gossypii*, the interest in producing riboflavin from LAB is due to the potential of fermented foods with high levels of riboflavin being produced in situ [100]. Mohedano et al. (2019) investigated five strains of *L. plantarum* as a probiotic by examining their ability to produce riboflavin as well as their capability to survive under digestive tract stresses. One strain of *L plantarum* at 3.33 mg/L survived well under gastric stress conditions, alluding to its potential as a probiotic strain for functional foods [96].

High yields of 5.72 mg/L riboflavin were obtained for another strain of *L. plantarum* by optimizing its growth medium. Glucose and sucrose were evaluated as carbon sources at both 30 °C and 37 °C, and the highest yields of riboflavin were observed when growing in a sucrose-based medium at 30 °C [99]. 

#### 2.3.2. Health Benefits of Riboflavin

A balanced diet will in most cases supply the necessary amount of riboflavin in healthy humans, as adult females need 1.1 µg/day while adult males need 1.3 µg/day [39]. Nevertheless, different population groups can be at risk of insufficient riboflavin supply such as the elderly, children, and pregnant women [101]. 

Riboflavin has been shown to exert an antioxidant effect in two ways: via prevention of lipid peroxidation and via the attenuation of reperfusion of oxidative injury [102]. The research conducted on this antioxidant activity in humans is, however, limited [103]. This antioxidant activity is attributed to the role riboflavin plays in the activity of several antioxidant enzymes such as superoxide dismutase, catalase, and glutathione peroxidase [102]. 

Some studies have also shown that intake of adequate amounts of riboflavin could lead to a decreased risk of breast cancer [104], lung cancer [105], colorectal cancer [106], gastric cancer [107], and ovarian cancer [108], emphasizing its importance in a healthy lifestyle. 

It has been hypothesized that riboflavin can have a neuroprotective effect on diseases such as Parkinson’s disease, migraine, and multiple sclerosis [103]. A low level of riboflavin has been reported in patients with Parkinson’s disease. By adding a riboflavin supplement to the diet and eliminating red meat, it was possible to increase motor functions [109]. This same effect has, however, not been observed in other studies [110,111]. 

There are a few studies that have investigated the effect of riboflavin on multiple sclerosis. In one study on animal models, riboflavin had a suppressive effect on neurological disability [112], but the same effect was not observed in a human interventional study [113]. 

Migraine is a common neurological disorder, estimated to affect around 3% in early childhood and up to 23% of the adult population [114]. Adults with a migraine who were administered 400 mg of riboflavin for 3 months or 6 months showed alleviation of migraine symptoms [115,116]. 

### 2.4. Menaquinone

There are two available forms of vitamin K: phylloquinone produced by plants, and menaquinone primarily produced by bacteria [68]. It is an essential cofactor involved in the post-transitional carboxylation found in proteins related to blood clotting, cardiovascular disease, and bone health [41]. *B. subtilis* is the most well-studied strain for menaquinone production, but some LAB are also capable of synthesizing menaquinone (Table 5) [117].

#### 2.4.1. Production of Menaquinone

The potential of synthesis of menaquinone by LAB was researched in a study that screened 21 strains. Of the screened species of LAB, five strains were selected due to their high production of menaquinone: three strains of *L. lactis* subsp. *Cremoris*, one strain of *L. lactis* subsp. *Lactis*, and one strain of *L. lactis*. All five of these strains were able to produce a meaningful amount of menaquinone when grown in non-fat dry milk [119]. 

*P. freudenreichii* was investigated both for its ability to produce menaquinone and its precursor 1,4-dihydroxy2-naphthoic acid (DHNA). When grown in a skim milk-based media in a 3 L bioreactor, relatively high yields of menaquinone of 0.12 mM were obtained [120]. 

#### 2.4.2. The Role of Menaquinone in Cardiovascular Disease and Bone Health

The daily requirements of menaquinone for adults vary between 55 and 65 µg/day according to the WHO [39].

Menaquinone has long been recognized for its role in blood coagulation but has also shown potential to improve bone health and inhibit the growth of cancer [121,122]. 

Studies have shown that the intake specifically of vitamin K in the form of menaquinone is associated with reduced coronary calcification and a reduced risk of cardiovascular disease [123,124,125]. The effects of vitamin K in the form of phylloquinone and menaquinone over 10 years in 4807 Dutch men and women over the age of 55 were investigated [123]. Interestingly, this study found that the intake of menaquinone but not phylloquinone reduced the risk of coronary heart disease and coronary calcification [123]. This comparison of phylloquinone to menaquinone was also performed in another study, which found a similar effect of only menaquinone reducing the risk of coronary calcification [126]. 

Menaquinone can play an important role in bone health as three different menaquinone-dependent proteins have been isolated from bone [124]. The effect of menaquinone on bone mineral density in human intervention studies is inconclusive, with some showing a beneficial effect [127,128] while another study has concluded no beneficial effect [129]. One major limitation of the study by Emaus et al. (2010) is, however, the short follow-up period of 1 year, with another study stating that effects might only become significant after 2 years of intervention [128]. In addition to improving bone mineral density, menaquinone has also shown indications of improving bone strength in a human intervention study [128].

## 3. Bacteriocins

Bacteriocins are antimicrobial peptides produced by certain bacteria, which can kill or inhibit the growth of both Gram-negative and Gram-positive bacteria (Table 6) [130]. Several applications of bacteriocins have been reported in the literature, including control of microflora in fermentation products [131] and extension of shelf life [132]. Bacteriocins are proving worthy of commercial interest, due to their preservation qualities, which can aid in fulfilling consumer demand for foods that are safe and long-lasting without the use of chemical preservatives [133,134].

Currently, the only commercially available bacteriocins are nisin produced by *L. lactis* and pediocin PA-1 produced by *Pediococcus* species [135]. These are both approved for safe use in food by the Food and Drug Administration (FDA) and the European Food Safety Authority (EFSA). In addition to nisin and pediocin, several other bacteriocins are highly studied but not yet approved for safe use in foods, including lactococcins, enterocins, and aureocins [136].

**Table 6:** Overview of nisin and pediocin produced by LAB. (MRS: De Man, Rogosa and Sharpe, TGE: Tryptone Glucose Extract, ICT: Immobilized Cell Technology).

Microorganism (s)	Product	Fermentation	Media	Yield	Ref.
*L. lactis*	Nisin	Flask	Skim-milk-based media	75 IU/mL	[137]
*L. lactis*	Nisin	Shake flask	Whey-based media	92.9 mg/L	[138]
*L. lactis*	Nisin	Shake flask	Whey-based media	1167 AU/mL	[139]
*L. lactis*	Nisin	Shake flask	Whey-based media	2618.7 IU/mL	[140]
*L. lactis*	Nisin	Single batch—free cells	Whey-based media	32,800 and 41,000 BU/mL (16,400 and 20,500 IU/mL)	[141]
*L. lactis*	Nisin	Single batch—free cells	MRS	160 AU/mL	[142]
*L. lactis*	Nisin	Repeated cycle batch—ICT	Whey-based media	20,480 IU/mL	[143]
*L. lactis*	Nisin	Fed batch	Complex	4185 IU/mL	[144]
*L. lactis*	Nisin	Fed batch	Whey	60.3 BU/mL	[145]
*L. lactis*	Nisin	Fed batch	Whey + glucose	124 BU/mL	[146]
*L. lactis*	Nisin	Fed batch	Whey + MRS nutrients	258.47 BU/mL	[147]
*L. lactis*	Nisin	Fed batch	Defined	2594 × 10^6^ IU/mL	[148]
*L. delbrueckii* subsp. *bulgaricus* *S. thermophilus*	Nisin	Continuous fermentation	Skim milk-based media	4500 BU/mL	[149]
*L. lactis* *K. marxianus*	Nisin	Single batch—free cells	Defined	98 mg/L (3920 IU)	[150]
*L. lactis* *S. cerevisiae*	Nisin	Shake flask	Defined	150.3 mg/L	[151]
*L. lactis* *P. acidilactici*	Nisin Pediocin	Shake flask	Whey-based media	74 BU/mL 195 BU/mL	[152]
*L. lactis* *P. acidilactici*	Nisin Pediocin	Shake flask	Whey-based media	9 BU/mL 45 BU/mL	[153]
*L. lactis* *P. acidilactici*	Nisin Pediocin	Shake flask	MRS Whey-based media	50 and 22.9 BU/mL 493.2 and 57.9 BU/mL	[154]
*L. lactis* *P. acidilactici*	Nisin Pediocin	Single batch—free cells	Whey-based media	3000 AU/mL (18 h) 1359 AU/mL (16 h)	[155]
*P. acidilactici*	Pediocin	Flask	Whey-based media	12,800 AU/mL	[156]
*P. acidilactici*	Pediocin	Flask	MRS	12,800 AU/mL	[157]
*P. acidilactici*	Pediocin	Flask	Whey-based media	150,000 AU/mL	[158]
*P. acidilactici*	Pediocin	Shake flask	Whey-based media	189 BU/mL	[159]
*P. acidilactici*	Pediocin	Shake flask	MRS Whey-based media	493.2 BU/mL 167.3 BU/mL	[160]
*P. acidilactici*	Pediocin	Single batch—free cells	TGE broth	40,000 AU/mL	[161]
*P. acidilactici*	Pediocin	Repeated cycle batch—ICT	MRS Whey-based media	4096 AU/mL (0.75 h) 4096 AU/mL (2 h)	[162]
*P. acidilactici*	Pediocin	Fed batch	Defined	712 BU/mL	[159]
*P. acidilactici*	Pediocin	Fed batch	Whey-based media	517 BU/mL	[160]
*P. acidilactici* *S. thermophilus* *L. delbrueckii* subsp. *bulgaricus*	Pediocin	Flask	Skim-milk-based media	6400 AU/mL	[157]
*P. acidilactici* *P. pentosaceus*	Pediocin	Flask	Whey-based media	3220 AU/mL 26,100 AU/mL	[163]
*P. pentosaceus* *L. plantarum*	Pediocin	Flask	Whey-based media	51,200 AU/mL	[164]

### 3.1. Production of Nisin

Nisin is commercially used in over 48 countries and has FDA and EFSA approval [135]. It was first isolated from milk in 1928 and gained approval as a safe food additive in 1969 by the Joint Food and Agriculture Organization/World Health Organization (FAO/WHO) [165]. Nisin is usually manufactured via fermentation of milk or whey using *L. lactis*. The fermentation broth is subsequently harvested, concentrated, separated, and spray-dried [130]. To reach the optimal nisin production, more complex media including MRS are usually required together with control over parameters such as pH and temperature [144]. 

Several studies have compared the nisin production yields from *L. lactis* grown on whey-based feedstocks with MRS media [145,154]. A selection of studies has reported that the production yields using MRS medium can reach 50 and 55 AU/mL [145,154], whereas typical yields of 22 and 22.5 AU/mL are achieved using diluted whey medium [145,154]. Malvido et al. (2019) supplemented a base whey medium with MRS nutrients. Nutrients were supplemented in concentrations corresponding to MRS media of 25%, 50%, 75%, 100%, and 125% w/v. The addition of MRS nutrients increased the nisin yield from 22.67 bacteriocin units (BU) for diluted whey to 57.26 BU for whey supplemented with 100 *v*/*w* of MRS nutrients. There was an observed increase in yield with MRS nutrient addition, but only up until 100%, where the yield stagnated [147]. 

It has been reported that to further optimize the production of nisin, it is necessary to provide additional protein in the form of, e.g., peptone, tryptone, or yeast extract [144]. 

To optimize nisin production, a fed-batch fermentation on a whey medium was studied with a feed composition of concentrated glucose (400 g/L), concentrated whey, and 4% yeast extract, and a feeding volume addition corresponding to the amount needed to restore initial total sugar concentration. Nisin production was increased to 50.6 and 60.3 BU/mL when compared to the yield obtained in batch cultures grown in whey of 22.5 BU/mL [154], and increased to 50 BU/mL when compared to growth batch culture growth in MRS [145].

The production efficiency and costs of production in fed-batch fermentation of nisin were compared in supplemented and unsupplemented whey. They had a feed consisting of concentrated whey and concentrated glucose and a feed profile adding volumes to the fermentation to restore the initial total sugar composition. The cost of nisin in a fed-batch fermentation supplemented with MRS nutrients was 30% lower than the cost of nisin in fed-batch fermentation with unsupplemented media. Here, the highest yield obtained on a whey-based fed-batch fermentation with MRS nutrients was 258 BU/mL compared to 124.66 BU/mL in a whey-based fed batch with no added nutrients [147].

### 3.2. Production of Pediocin

Pediocin is a bacteriocin with a broad spectrum, meaning that it is capable of inhibiting several different species of Gram-positive bacteria [166]. It is, however, particularly known for its ability to inhibit the growth of *L. monocytogenes* and *S. aureus,* which are both common food pathogens [167]. Both *P. acidilactici* and *P. pentosaceus* are capable of pediocin production (Table 6), are well-known from food fermentations, and are either naturally present or added as a starter culture in the fermentation of vegetables and sausages [166,168].

The influence of the different salts ammonium phosphate, calcium chloride, potassium dihydrogen phosphate, and manganese(II)sulphate monohydrate on pediocin production was tested in a direct plate bioassay for rapid assessment. Manganese is an essential growth factor for lactic acid bacteria, and the addition of MgSO_4_ also resulted in a significantly increased pediocin yield. Ammonium phosphate, calcium chloride, and potassium dihydrogen phosphate each resulted in a suppression of pediocin production [142]. A whey and yeast-extract-based media could, therefore, possibly be further optimized with the addition of manganese. The growth and pediocin production of Pediococcus have also been tested in both a repeated batch and a fed-batch setup with whey and yeast extract as the batch media, with reports of increased yield [159,160,162]. 

Pediococcus species more readily metabolize glucose than other carbon sources [142,155]. Excessive glucose in the fermentation broth can, however, have an inhibitory effect, and its addition in a simple batch resulted in decreased pediocin, suggesting substrate inhibition. The addition of glucose in the feed of a fed-batch fermentation instead resulted in great improvements in the pediocin yields, showing at least a two-fold increase of 517 BU/mL in a fed-batch compared to 167 BU/mL in a simple batch in one study [160]. Pediocin has, furthermore, been determined to be a pH-dependent metabolite, and pH should be maintained at levels lower than 5 to achieve maximum production [153,160,169]. This is due to a requirement of low pH for the post-translational processing of pre-pediocin to active pediocin [161]. 

### 3.3. Mixed Culture Induction

The presence of other bacterial strains can enhance bacteriocin production either via the production or consumption of metabolites or by acting as a stress signal, which, in some cases, increases bacteriocin production. Pediocin production by *P. pentosaceus* was increased by 250% in the presence of *L. plantarum* [164], while another study observed that the co-culturing of *P. acidilactici* with *S. thermophilus* and *L. delbrueckii* enhanced pediocin production in fermented milk [157].

*S. cerevisiae* improved nisin production in a study by Shimizu et al. (1999) from *L. lactis* by 85% to a final yield of 150.3 mg/L. This was attributed to the lactic- and acetic acid assimilation by *S. cerevisiae,* which normally acts as a limiting factor for nisin production [151]. A similar enhancement of 70% was observed when *L. lactis* was co-cultured with another yeast, *K. marxianus*, where it was also concluded that the increased nisin production was caused by lactic acid assimilation and the resulting control of pH levels [150].

### 3.4. Health Benefits of Bacteriocins

Bacteriocins produced by LAB possess several health benefits. Their antimicrobial activity can be applied in the treatment of harmful bacteria, as they can selectively target pathogenic organisms, while not affecting commensal bacteria. This makes them a valuable alternative to antibiotics that are less target-specific [170].

Additionally, research is suggesting that bacteriocins can have applications in the treatment of cancer as well as immunomodulatory effects (Figure 2).

#### 3.4.1. Treatment of Pathogens and Alternatives to Antibiotics

The anti-listerial effect of both purified pediocin and its producer strain *P. acidilactici* was investigated in vivo in mice infected with *L. monocytogenes*. The effect of oral administration of *P. acidilactici* did not result in a decrease in *L. monocytogenes* in the intestine, liver, or spleen, while the administration of purified pediocin resulted in a 2-log decrease in *L. monocytogenes*. None of the treatments altered the composition of the gut microbiota, making purified pediocin a promising agent against *L. monocytogenes* [171]. In this study, however, administration of *P. acidilactici* was only given as a single dose, and purified pediocin was administered over 3 days. Another study administered both a pediocin and non-pediocin strain of *P. acidilactici* and a nisin-producing strain of *L. lactis* for 16 days in mice infected with vancomycin-resistant enterococci (VRE). The results showed that, on day 6, none of the infected mice administered either pediocin-producing *P. acidilactici* or nisin-producing *L. lactis* had detectable levels of VRE [172]. 

*S. aureus* is one of the most common pathogens in the upper respiratory tract, and nisin has been assessed for its ability to combat *S. aureus* infections in vivo in immunocompromised rats. Rats were provided nisin intranasally, and growth of *S. aureus* was inhibited [173]. These results suggest that nisin and pediocin can indeed be used to fight pathogens and could have importance against pathogens with resistance to antibiotics. Another advantage of bacteriocins over antibiotics is their specific activity, which does not affect the commensal bacteria in the gut, as antibiotics do [174]. A study investigated the sensitivity of 21 common intestinal bacteria to pediocin and two types of nisin in vitro. Both types of nisin inhibited all the Gram-positive bacteria and only one of the Gram-negative bacteria. Pediocin, on the other hand, did not inhibit any of the 21 intestinal bacteria assayed, which further strengthens its potential as a non-invasive alternative to pathogenic infections [174].

#### 3.4.2. Anti-Cancer Treatment

There is research that suggests the cytotoxicity of bacteriocins against cancer cells [175]. 

Nisin has been reported to have cytotoxic and antitumor effects against head and neck squamous cell carcinoma in vivo in mice [176]. Mice were fed 200 mg/kg of nisin for three weeks, which resulted in a significant reduction in tumor size compared to the control. These effects of nisin were also tested in vitro and were found to be due to induced apoptosis, cell cycle arrest, and reduction in cell proliferation [176]. This effect of nisin on head and neck squamous cell carcinoma was also observed in vivo in another study in mice [177]. It was also found that when receiving dosages of nisin, the tumor size would reduce and would prolong survival. 

Nisin has also shown possible application potential as an adjunct together with the chemotherapeutic agent doxorubicin in the treatment of skin cancer [178]. Carcinogenic mice were treated with either nisin, doxorubicin, or a combination of the two. The tumor size was reduced by 14% with nisin, 51% with doxorubicin, and 66.82% when mice were treated with both agents, as compared to untreated groups. 

Pediocin has also shown potential as an anti-cancer agent in vitro against various cell cancer lines such as colon, liver, cervical, and mammary gland cancer cells [179,180]. 

#### 3.4.3. Immunomodulatory Role of Bacteriocins

Host-defense peptides are ubiquitous and play an important role in the innate immune system. Despite being smaller and having a different structure, bacteriocins share similar physiochemical properties to host-defense peptides, which could indicate that they might have similar immunomodulatory properties [181,182]. This immunomodulatory ability has been demonstrated in vivo in mice [183] and turbot [184]. By feeding mice nisin in the commercial form Nisaplin, an increase in the T-lymphocytes CD4 and CD8 and a decrease in B-lymphocyte levels were observed. Long-term administration resulted in a return to normal levels of B- and T-lymphocytes and an increase in the macrophage/monocyte population [183]. 

An in vitro study found that nisin could induce the stimulation of the chemokines monocyte chemoattractant protein-1 (MCP-1), Gro-α, and IL-8 while significantly reducing TNF-α in response to bacterial lipopolysaccharide in human peripheral blood mononuclear cells [182].

Lastly, a study found that nisin was able to activate neutrophils and suggested that nisin might be capable of influencing multiple subsets of host immune cells [185]. 

The research conducted on nisin’s ability to alter the host immune response is limited, so further studies in this area are needed.

## 4. Bioactive Peptides

Proteins that occur naturally in food can, in addition to providing nutritional benefits, also contain sequences called bioactive peptides that can exert various health benefits (Figure 2) [186]. When present in the parental protein, these sequences are inactive but can be released in three ways: through hydrolysis by gastrointestinal enzymes, through the action of proteolytic enzymes derived from plants or microbes, or by microbial fermentation with microbes exerting proteolytic activity [187]. The focus in this section will solely be on the bioactive peptides released from microbial fermentation. During the fermentation of milk, LAB are capable of hydrolyzing milk proteins to make nitrogen sources like peptides and amino acids available [188]. 

### 4.1. Angiotensin-Converting Enzyme Inhibitory Peptides

Several members of the species Lactobacillus, Bifidobacterium, and Pediococcus are described in the literature as angiotensin-converting-enzyme-inhibitory peptide (ACE-inhibitory peptide) producers [189,190,191] (Table 7). Functional dairy products containing ACE-inhibitory peptides such as fermented milk [192,193,194,195] and cheese [196,197] have been developed. These products are characterized by a high degree of proteolysis, whereas products with a lower proteolytic activity such as yoghurt, fresh cheese, and quark have a lower ACE-inhibitory activity [198]. 

Unfermented whey already contains a certain amount of ACE-inhibitory peptides, but by fermenting it with the microbiota naturally present in cheese whey, the ACE-inhibitory activity increases from 22% in unfermented whey to 60–70% in fermented whey [203]. Another study by Ahn et al. (2009) showed that a further supplementation of whey with glucose and yeast extract led to an increase in the biomass, thereby increasing the proteolytic activity and, in turn, leading to a higher yield of peptides [190]. Here, *L. brevis*, *L. helveticus,* and *L. paracasei* were used for peptide production, with the peptides derived from whey fermentation of *L. helveticus* showing high inhibitory activity, with IC50 values of 5.3 and 7.8 µg/mL. 

*P. acidilactici* is another species that has also been shown to exhibit good ACE-inhibitory activity, as was found by screening 34 different strains of LAB grown in a whey-based medium [189]. *P. acidilactici* had an ACE-inhibitory activity of 84.7% with an IC50 of 19.78 µg/mL. 

Sodium caseinate was used to produce a fermentate with ACE-inhibitory activity by applying *L. animalis* [204]. The fermentate had an 85.51% ACE-inhibitory activity with an IC50 value of 8 µg/mL, which is only a slightly higher dose needed than the commercially available captopril, which demonstrates inhibitory activity at a dose of 5 µg/mL [204]. Raveschot et al. (2020) compared the ACE-inhibitory peptide productivity of *L. helveticus* in three different fermentation setups: a simple batch, a continuous bioreactor, and a continuous membrane bioreactor (MBR). The mean productivity of the MBR setup was 0.7 g/L/h, which was comparable to that of the simple batch of 0.33 g/L/h, but higher than the continuous bioreactor, which had a mean productivity of 0.103 g/L/h. The specific productivity was also calculated as a function of bacterial biomass, where the MBR setup was superior to the other setups with a rate of 15.8 g/g compared to 9.99 g/g and 7.13 g/g in the batch and continuous batch, respectively. In that study, a fermentate with an IC50 value of 0.47 mg/L for *L. helveticus* was obtained [205]. 

#### Antihypertensive Effect and Cardiovascular Diseases

Drugs that inhibit ACE are common for the treatment of hypertension. ACE is needed for the conversion of angiotensin 1 to angiotensin 2, which narrows the blood vessels and can cause higher blood pressure [206]. 

The blood-pressure-lowering effects of ACE-inhibitory peptides have been demonstrated in several studies with subjects suffering from hypertension [207,208,209]. Ingestion of tablets of milk fermented with *L. helveticus* was administered for 4 weeks in subjects with either high-normal blood pressure or mild hypertension. For both groups, a lowered blood pressure was observed for subjects ingesting the tablets compared to a placebo group. The placebo tablets had a similar composition to the test-group tablets, but they had no ACE-inhibitory peptides, indicating the blood-pressure-lowering effect was due to the presence of the peptides [207]. 

Another proposed effect of milk fermented with *L. helveticus* is the alleviation of arterial stiffness. Long-term administration of the milk-containing peptides showed a significant reduction in arterial stiffness compared to the placebo group [210]. That study also investigated the improvement of endothelial function but found no effects from the peptide-containing milk. Another study did, however, observe an improved vascular endothelial function when administering peptides to hypertensive subjects [211]. This mechanism was determined to be related to the enhanced production of vasodilating substances. 

### 4.2. Peptides with Antioxidant Activity

Some LAB possess the ability to excrete peptides with antioxidant activity (Table 8). These peptides can decrease the risk of accumulation of reactive oxygen species (ROS) as well as degrade superoxide anions and hydrogen peroxide [212,213].

Virtanen et al. (2007) screened 25 LAB strains for their ability to exhibit antioxidant activity in milk media [216]. While all strains had antioxidant activity to some degree, six strains had a higher inhibition rate: 2 *Lc. cremoris*, *L. lactis*, *L. jensenii*, *L. acidophilus*, and *L. helveticus*. A correlation between the degree of hydrolysis and radical scavenging activity was found for all strains, with an additional correlation of bacterial growth with radical scavenging activity found for *L. acidophilus* and *L. helveticus*. Lipid peroxidation inhibitory activity was found to be more related to bacterial growth than to proteolysis. When two or three of the strains were cultivated together, this increased antioxidant activity [216]. This mixed-strain approach to achieving a higher antioxidant activity was used in another two studies co-cultivating yoghurt bacteria with lactobacilli (e.g., [214]).

The traditional yoghurt-fermenting strains *S. thermophilus* and *L. delbrueckii* subsp. *bulgaricus* were used to produce yoghurt and test for its potential antioxidant activity. That study concluded that yoghurt can indeed have potential as a natural antioxidant [213].

Another study also used the yoghurt strains but supplemented them with additions of *L. casei*, *L. paracasei*, and/or *L. acidophilus* to enhance the antioxidant activity of yoghurt. The yoghurt-fermenting strains alone demonstrated good antioxidant activity, but the addition of the lactobacillus strains resulted in a lowering of the IC50 value. The highest antioxidant activity observed was when all three strains of lactobacillus were added [214], and the same was found in another study by Lin and Yen (1999) [213]. 

*L. casei* was used in a study to investigate the influence of several nutrients on the production of antioxidant peptides in goat milk fermentation [215]. It was found that the addition of casein peptone, glucose, and calcium lactate had significant positive effects on the antioxidant activity. The effects of glucose and calcium lactate are most likely caused by stimulation of *L. casei* growth. Casein peptone could increase the release of peptide, which was also observed in another study for caseinate [217]. 

#### Health Benefits of Antioxidant Peptides Produced by LAB

Some of the antioxidant potential in fermented dairy products is considered to be caused by the high release of antioxidant peptides by the proteolytic systems in many LAB [218]. In vivo studies in either animal or human models demonstrating the specific effect of antioxidant peptides are scarce [218]. One study fed ageing rats with either unfermented milk or milk fermented with *L. fermentum* and observed that after 2 months, the numbers of the antioxidant enzymes superoxide dismutase, catalase, and glutathione peroxidase were higher in the liver cells of rats fed the fermented milk compared to the unfermented milk [219]. The influence of fermented and unfermented milk on a weanling rat model was also compared in another study [220]. The ability to reduce lipid peroxidative stress was evaluated, but no difference between the two milk types was observed. An antiperoxidative effect was still observed when compared to the control, indicating the milk protein rather than the lactobacillus to be responsible for this effect. Both of the above-mentioned results were also demonstrated in a third study on rats fed either fermented or unfermented whey. An increase in antioxidant enzymes and antiperoxidative activity was observed for both fermented and unfermented whey, although this study did observe peroxidative changes to be more pronounced in fermented whey [221].

## 5. Bioactive Compounds

Some species of LAB have gained attention for their ability to produce the bioactive compounds gamma-aminobutyric acid (GABA) and carotenoids [222,223]. GABA is a neurotransmitter with known health benefits such as its ability to calm the nervous system while carotenoids are pigments with known antioxidant activity (Figure 2) [224,225]. 

### 5.1. Gamma-Aminobutyric Acid

γ-aminobutyric acid (GABA) is a bioactive amine that has been shown to confer health benefits. It acts as a neurotransmitter in the nervous system and can lower blood pressure in mildly hypertensive patients [226], and it has also been suggested to have anti-tumor effects [227]. It is naturally present in several foods such as tomatoes, teas, soybeans, and fermented foods and can, therefore, be obtained naturally through the diet, but much higher concentrations are obtainable through LAB fermentations (Table 9) [228]. 

#### 5.1.1. Production of GABA

The pH value during the fermentation plays an important role both in the cell growth of LAB and in their production of GABA. The synthesis of GABA is dependent on the enzyme glutamic acid decarboxylase (GAD), which acts as a catalysator, and pH is, therefore, an important factor when considering yields of GABA [257,260]. The importance of maintaining a pH value between 4.5 and 5 has been reported in several studies that have documented optimal GABA synthesis when pH was kept at this level [229,230,231,241,244,257]. A pH value of 6 did result in higher biomass in a study cultivating *L. brevis*, but even with the higher biomass, the yield of GABA was still higher at pH 5 [231]. 

GABA production itself is not affected by lower temperatures of around 30 °C but can lead to lower yields of biomass, which, in turn, lead to less total yield of GABA [229,231]. Higher temperatures above 40 °C did support growth but were inhibitory to GABA production [229,230,231,246]. The optimal temperature for both cell growth and GABA synthesis is, therefore, within the range of 35–40 °C. 

Due to the different optima for cell growth and GABA synthesis, a two-stage fermentation was developed for *L. brevis*. Cultures were maintained at 35 °C and a pH value of 5 for the first 32 h to stimulate cell growth, and they were subsequently adjusted to 40 °C and a pH of 4.5. This resulted in an increase in GABA from 398 mmol/L in the one-stage fermentation to 474.70 mmol/L in the two-stage fermentation [232]. 

Another factor that can increase GAD activity is the addition of pyridoxal-5′-phosphate (PLP), which acts as a coenzyme. Studies have reported an increase in the GABA yield of *L. paracasei* from 200 mM without PLP to 300 mM with PLP [244], and in *S. thermophilus* fermentation, from 6 g/L without PLP to 8 g/L with PLP addition after 24 h [257]. Other studies did, however, not observe any change in yield in *L. brevis* fermentations, but that was suggested to be due to sufficient synthesis in the fermentation broth [231,246]. 

Monosodium glutamate (MSG) is a stimulant of GABA production, but if it is present at levels too high, it has been shown to have inhibitory effects [233,257]. In *L. plantarum* cultivation, GABA synthesis was significantly influenced by the addition of 100 mM of MSG in the fermentation broth, as this induced an increase in production by 7.7 to a value of 721.35 mM [261]. This stimulatory effect was similarly observed for *L. brevis* within a range of 2.25% and 4–6% *v*/*w* [230,247], for *S. thermophilus* within a range of 10–20 g/L [257], and for *L. lactis* and *L. plantarum* at 1% *v*/*w* [246]. 

A strategy to overcome the inhibitory effects of high MSG concentrations is to supplement the feed with it in fed-batch fermentations [231,232]. In a study by Peng et al. (2013), an initial concentration of 100 mmol/L MSG was added to a defined medium consisting of glucose, yeast extract, peptone, sodium acetate, and ions. The feed consisted of 106 mmol of MSG and was added at 32 h and 56 h. By the end of fermentation, the final yield of GABA was 526.33 mmol/L. The yield was only slightly higher than without the feed (474.79 mmol/L), but due to the additional increase in volume, a much higher total GABA productivity was obtained [232].

To a fermentation broth consisting of glucose, peptone, tween80, and MnSO_4_, 400 mM of glutamate was added. It was found that concentrations exceeding 500 mM strongly inhibited cell growth. The feed was added at 12 h and 24 h and consisted of 280.8 g and 224.56 g of glutamate. This feeding strategy was detrimental to the cell growth, but highly effective for a high yield of GABA in a short fermentation time, as it yielded 1095 mM [231]. 

A very high yield of 205.8 g/L GABA by *L. brevis* was also produced in a medium with 295 g/L of L-glutamic acid [234]. The issue with L-glutamic acid or glutamate is, however, the high production cost, making it less feasible for cost-effective production [247]. 

Co-culturing microbes is another efficient approach to increase the yield of GABA and has been successfully applied in many studies. Fermentation of milk with a combination of a strain with high proteolytic activity together with a GABA-producing strain has led to efficient GABA production, together with additional reports of a shortened fermentation time [240,254,259]. The proteolytic strains are capable of breaking down milk proteins into peptides, which can then be used as substrates by the GABA-producing strain. 

Co-culturing of LAB and fungi has been reported with combinations of *L. plantarum* and *Ceriporia lacerate*, leading to the production of 15.53 g/L while the combination of *Lactobacillus futsaii* and *Candida rogusa* led to a GABA productivity of 135 mg/L/h [262,263]. 

Whey could be a suitable and low-cost option for GABA production by LAB and has been investigated as a substrate [238,254]. A GABA yield of 553 mg/L was obtained via cultivation of *L. brevis* by utilizing a media consisting of 14.95% whey and 4.95% MSG [238]. A higher GABA yield of 3.65 g/L was obtained in whey fermentations of a co-culture of *L. plantarum* and *L. lactis* subsp *lactis*. In that study, soy protein hydrolysate was used as a source of glutamic acid, and when added to the whey, the yield increased from 1 g/L to 3.65 g/L [254]. Both of these studies utilized very simple media for GABA production on whey and did not use pH control or the addition of MSG, PLP, or tween80. It is possible that these additions and a potential fed-batch fermentation setup could further increase the yield of GABA. 

#### 5.1.2. Health Benefits of GABA

GABA has a wide variety of different health benefits including a hypotensive effect, action as a neurotransmitter, and treatment of diabetes, cancer, and asthma [264]. 

The hypotensive effect of GABA is similar to that of ACE-inhibitory peptides, i.e., it works by inhibiting noradrenalin release, which inhibits perivascular nerve stimulation and thereby facilitates a hypotensive effect [265]. Spontaneously hypertensive rats (SHR) and normotensive Wistar–Kyoto rats (WKY) were given GABA to investigate its blood-pressure-lowering capabilities. GABA was given as a single dose of 0.5 mg/kg and showed a significant lowering of the systolic blood pressure in SHR but not in WKY rats [265]. This was confirmed in the same two rat models in a later study dosing GABA either in the pure form or in an enriched fermented milk product [266]. In that case, a long-term dosage was also investigated and a significantly slower increase in blood pressure of both rat models was observed for those administered either GABA or GABA-enriched fermented milk compared to controls [266]. Milk fermented with the LAB *L. casei shirota* and an *L. lactis* containing GABA was tested in mildly hypertensive patients [226]. Blood pressure was significantly decreased in patients after 2 to 4 weeks and remained decreased through all 12 weeks [226]. 

Disruption in the GABA receptor expression has been associated with anxiety spectrum disorders and has also been shown to have a role in mood disorders like depression [267]. Administration of *L. rhamnosus* to mice modulated the GABA receptors, and mice showed a behavior that was less anxious, stress-related, or depressive compared to the control group [268]. This stress-reducing effect of GABA has also been demonstrated in humans through drinking GABA-enhanced water [269] and via ingestion of GABA-enriched chocolate [270].

When consuming a powdered preparation of GABA, an improvement in sleep was observed through reduced sleep latency as well as an increase in non-rapid eye movement sleep in healthy adults [271]. For a group of patients suffering from chronic fatigue, the ingestion of a GABA-enhanced drink before solving an arithmetic task resulted in reduced occupational fatigue afterward [272].

A study suggested GABA as a tool to prevent obesity induced by a high-fat diet. It was demonstrated that GABA improved oxidative stress and thyroid function, and that treatment with GABA in high-fat-diet mouse models prevented weight gain [273]. 

GABA also has potential as a therapeutic against diabetes and has shown an inducing effect on the secretion of insulin [274], and it can even reverse established diabetes in a diabetic mouse model [275]. Based on the strong in vivo data of GABA in diabetes, the administration of a food product with GABA present serves as a promising tool in the treatment and prevention of diabetes. 

Lastly, GABA could be used as an anti-cancer agent that acts by having tumor-suppressing activity in lung adenocarcinoma cells [276] and inhibiting cell proliferation in colon cancer cells [277]. Mouse and human leukemia cells as well as human cervical cancer cells were treated with germinated rice enhanced with GABA. In that study, GABA had an inhibitory action on leukemia cell proliferation and also showed a stimulatory action on cancer cell apoptosis [278]. 

### 5.2. Carotenoids

Carotenoids are bioactive molecules that naturally occur in most fruits and vegetables. They can be produced by a wide range of organisms such as plants, algae, fungi, yeast, and bacteria. Humans, however, are unable to synthesize carotenoids and depend on intake through the diet [223]. Intake of carotenoids is associated with many health benefits, primarily by exerting antioxidant activity [225]. Several carotenoids are produced industrially and can be used both as a source for fortification of foods and as colorants [279]. The majority of studies on the biotechnological production of carotenoids are from yeasts, with fewer reports of synthesis through bacterial production [280,281]. The major challenge surrounding bacterial production of carotenoids is the lower yields when compared to other microorganisms, and developing industrially relevant strains is, therefore, essential to overcoming this issue [223,282]. 

#### 5.2.1. Production of Carotenoids

The research conducted on the production of carotenoids by LAB is sparse as yeasts are preferred due to higher yields; however, some research has been conducted investigating this trait in LAB (Table 10). In one study, 158 different strains of LAB were screened for their ability to produce carotenoids, and out of these, only 36 produced the pigment. Specifically, strains belonging to *L. plantarum*, *L. fermentum*, and *P. acidilactici* had this ability. All these strains did, however, produce carotenoids in low amounts, with a strain of *L. fermentum* yielding the highest amount at 765 µg/kg CDW [283]. 

Garrido-Fernández et al. (2010) cultivated *L. plantarum* in four different brands of MRS as well as two defined media and were able to obtain 54.55 mg/kg of cell dry weight in a defined medium. The effect of length of cultivation was tested by sampling at 24 h, 48 h, 72 h, and 96 h, but a decrease in carotenoid concentration in the broth was observed over time, and 24 h was selected as the optimum cultivation time [284]. *L. plantarum* was also examined in another study that used a Plackett–Burman approach to media optimization and used date syrup as a carbon source, which had a final maximum yield of 54.89 mg/kg of cell dry weight [285]. 

It has been found that the cultivation of LAB under aerobic conditions is essential for optimal carotenoid production as it increases the expression of genes in carotenoid biosynthesis [286]. 

A research group investigated the co-culturing of LAB with the yeast *R. rubra* [287,288,289,290]. These studies, however, used *R. rubra* as the main carotenoid producer, with the LAB added to the culture to create an optimal growth environment for *R. rubra*.

**Table 10:** Summary of carotenoid-producing strains and yields.

Microorganism (s)	Fermentation	Medium	Yield	Ref.
*L. fermentum*	Flask	MRS	765 µg/kg CDW	[283]
*L. plantarum*	Flask	Supplemented MRS	54.89 mg/kg CDW	[285]
*L. plantarum*	Flask	Defined medium	54.55 mg/kg CDW	[283]
*L. casei* *R.rubra*	Flask	Supplemented whey ultrafiltrate	12.35 mg/mL	[287]
*L. delbrueckii.* subsp. *bulgaricus* *S. thermophilus* *R. rubra*	Flask	Supplemented whey ultrafiltrate	13.09 mg/mL	[288]
*L. delbrueckii.* subsp. *bulgaricus* *S. thermophilus* *R. rubra*	Flask	Supplemented whey ultrafiltrate	13.37 mg/L	[289]
*L. helveticus* *R. glutinis*	Batch	Supplemented whey ultrafiltrate	8.3 mg/L	[290]

#### 5.2.2. Health Benefits of Carotenoids

Carotenoids play an important role in human nutrition as they can act as a precursor for the synthesis of vitamin A [291]. Although vitamin A is naturally present in many food products such as dairy, fish, orange and yellow vegetables, and leafy greens, deficiencies of vitamin A are still a prominent issue in some developing countries [292].

Low-density lipoprotein (LDL) is linked to the pathogenesis of atherosclerosis. Healthy adults consumed a carotenoid supplement in various concentrations, and it was found that a daily concentration over 3.6 mg of a carotenoid led to an inhibition of LDL oxidation, thereby having potential as a parameter for the prevention of atherosclerosis [293]. Carotenoid supplementation also showed an effect in the improvement of lipid profiles. Non-obese subjects with mild hyperlipidemia were given a dose of either 0, 6, 12, or 18 mg of carotenoids per day for 12 weeks. BMI and LDL levels remained unaffected, but triglyceride levels decreased significantly with doses of 12 and 18 mg/day, and levels of high-density lipoprotein (HDL) increased significantly in doses of 6 and 12 mg/day [294]. Long-term supplementation of a carotenoid did, however, not prove to impose any effects on the development of cardiovascular disease or mortality associated with cardiovascular disease [295,296]. Human trials have, furthermore, demonstrated a positive effect of dietary supplementation of carotenoids in weight management and obesity [297,298,299].

In vitro and in vivo studies have indicated that carotenoids stimulate bone formation and bone cell proliferation while also having an inhibitory effect on bone resorption [300,301,302,303]. In human trials, the intake of carotenoids over a 4-year period was also associated with a beneficial effect on bone health, and the results showed a protective role of carotenoids for bone mineral density [304].

It has been suggested that the antioxidant effect of carotenoids could have a preventative effect and act as a treatment for Alzheimer’s disease [305], but a study has found no effect of carotenoids on the decreased risk of Alzheimer’s disease and dementia [306].

The effects of carotenoid supplementation on the prevention of cancer show inconclusive results, with some studies claiming a reduction in cases [307,308] while others observed no effect [309,310].

## 6. Conclusions

The introduction of novel functional products on the European market can be a challenging process as claims related to health need to be scientifically substantiated and evaluated in a process that can be complicated for producers to navigate. This extensive framework of regulations does, however, simultaneously ensure trust from consumers in new food products. With an increasing demand and interest in fortified fermented dairy products, an in-depth understanding of the possibilities and challenges of the production of bioactive ingredients is crucial.

This review can function as a reference for future research aiming at producing functional dairy foods utilizing LAB-based fermentations, by providing a comprehensive overview of previously conducted research on the production of bioactive ingredients.

LAB are an essential constituent of a wide range of fermented products, which are already an integral part of the human diet. Here, they enhance the nutritional, organoleptic, and functional properties of the products. Understanding the different bioactive ingredients such as vitamins, bacteriocins, bioactive peptides, and bioactive compounds excreted during fermentation by LAB can aid in the targeted development of dairy products with desired health claims. This includes the selection of appropriate strains, optimized media composition, and suitable bioprocess parameters.

Consumers are increasingly seeking products that offer additional health benefits to nutrition. Exploring the potential and understanding the production processes of the bioactive ingredients produced by LAB are, therefore, important as they can further drive forward the innovation and development of the functional food industry market.

Employing molecular approaches such as various omics technologies (transcriptomics, metabolomics, genomics, and proteomics) can aid in furthering our understanding of the underlying mechanisms of LAB. Additionally, the implementation of digital tools can optimize the volumetric productivity, reduce the cost, and increase the reliability and the quality of bacterially produced bioactive ingredients [311].

## Figures and Tables

**Table 2:** Overview of folate-producing lactic acid bacteria (MRS: De Man, Rogosa, and Sharpe).

Microorganism (s)	Fermentation	Medium	Yield	Ref.
*L. acidophilus*	Flask	MRS	37.2 µg/L	[47]
*L. amylovorus*	Flask	MRS	81.2 µg/L	[47]
*L. amylovorus* *S. thermophilus* *L. delbrueckii* subsp. *bulgaricus*	Flask	Milk	263 µg/L	[47]
*L. brevis*	Flask	Supplemented whey permeate	131 µg/L	[48]
*L. casei*	Flask	MRS	1.5 µg/L	[47]
*L. coryniformis*	Flask	MRS	81 µg/L	[49]
*L. delbrueckii* subsp. *bulgaricus*	Flask	MRS	54 µg/L	[50]
*L. delbrueckii* subsp. *bulgaricus* *S. thermophilus*	Flask	Non-fat milk	180 µg/L	[51]
*L. fermentum*	Flask	MRS	6.9 µg/L	[47]
*L. fermentum*	Flask	Supplemented whey permeate	84 µg/L	[48]
*L. helveticus*	Flask	MRS	89 µg/L	[50]
*L. lactis*	Flask	MRS	45 µg/L	[50]
*L. lactis* subsp. *cremoris*	5 L batch bioreactor	Skim milk powder	187 µg/L	[40]
*L. lactis* subsp. *lactis*	Flask	M17	291 µg/L	[50]
*L. lactis* subsp. *lactis biovar diacetylactis*	Flask	M17	100 µg/L	[50]
*L. paracasei* subsp. *paracasei*	Flask	MRS	38.7 µg/L	[47]
*L. plantarum*	Flask	MRS	57.2 µg/L	[47]
*L. plantarum*	Flask	Supplemented whey permeate	397 µg/L	[48]
*L. plantarum*	Flask	MRS	108 µg/L	[49]
*L. plantarum* *P. freudenreichii*	1 L bioreactor	Supplemented whey permeate	8399 µg/L	[52]
*L. reuteri*	Flask	Supplemented whey permeate	125 µg/L	[48]
*L. sakei*	Flask	MRS	107 µg/L	[49]
*S. thermophilus*	Flask	M17	202 µg/L	[50]
*S. thermophilus*	2 L batch bioreactor	Modified M17	54.53 µg/L	[53]

**Table 3:** Overview of cobalamin-producing Propionibacteria.

Microorganism (s)	Fermentation	Media	Yield	Ref.
*P. acidipropionici*	1 L batch	Complex media	3.3 mg/L	[70]
*P. denitrificans*	120 L Batch	Complex media	177.49 µg/L	[71]
*P. denitrificans*	120 L Batch	Complex media	214.3 µg/mL	[72]
*P. freudenreichii*	5 L batch	Defined media	9.45 µg/mL	[73]
*P. freudenreichii*	Cell recycle system	Complex media	24.93 µg/mL	[74]
*P. shermanii*	Flask	Whey based medium	8.43 µg/L	[75]
*P. shermanii*	Flask	Whey based medium	2.97 µg/L	[76]
*P. shermanii*	Flask	Whey based medium	4.51 µg/L	[77]

**Table 4:** Overview of riboflavin-producing microorganisms (CDM: chemically defined media, MRS: De Man, Rogosa, and Sharpe).

Microorganism (s)	Fermentation	Medium	Yield	Ref.
*L. acidophilus*	Flask	Whey-based medium	2.93 mg/L	[93]
*L. fermentum*	Flask	CDM	1.2 mg/L	[94]
*L. fermentum*	Flask	CDM	3.49 mg/L	[95]
*L. lactis*	Flask	Whey-based medium	2.61 mg/L	[93]
*L. plantarum*	Flask	MRS	3.33 mg/L	[96]
*L. plantarum*	Flask	Supplemented MRS	3.33 mg/L	[97]
*L. plantarum*	Flask	Optimized MRS	12.33 mg/L	[98]
*L. plantarum*	Flask	CDM	5.72 mg/L	[99]

**Table 5:** Overview of menaquinone-producing LAB.

Microorganism (s)	Fermentation	Media	Yield	Ref.
*L. fermentum*	Flask	Rogosa medium Skim milk	184 µg/L 63.93 µg/L	[118]
*L. lactis* subsp. *cremoris*	Flask	Milk-based media	534 nmol/g of cells	[119]
*L. lactis* subsp. *lactis*	Flask	Milk-based media	717 nmol/g of cells	[119]
*Leu. lactis*	Flask	Milk-based media	173 nmol/g of cells	[119]
*P. freudenreichii*	3 L fermenter	Milk-based media	0.3 mM	[120]

**Table 7:** Summary of LAB producing ACE-inhibitory peptides and their yields (MRS: De Man, Rogosa, and Sharpe).

Microorganism (s)	Fermentation	Media	ACEI Ability	IC50	Ref.
*L. acidophilus*	Flask	Skim-milk-based media	-	730 µg/mL	[195]
*L. brevis*	Flask	MRS	79.03	1280 µg/mL	[189]
*L. brevis*	Flask	Whey-based media	64.7	1130 µg/mL	[190]
*L. casei*	Flask	Skim-milk-based media	-	250 µg/mL	[195]
*L. casei*	Flask	Skim-milk-based media	100		[199]
*L. casei*	Flask	Goats-milk-based media	34.3	-	[200]
*L. delbruckii* subsp. *bulgaricus*	Flask	Skim-milk-based media	-	780 µg/mL	[195]
*L. helveticus*	Flask	Whey-based media	84.2	860 µg/mL	
*L. helveticus*	Flask	Skim-milk-based media	-	1460 µg/mL	[195]
*L. helveticus*	Flask	Skim-milk-based media	67.18	-	[201]
*L. helveticus*	Flask	Goats-milk-based media	51.3	-	[200]
*L. paracasei*	Flask	Whey-based media	63.9	1130 µg/mL	[190]
*L. plantarum*	Flask	MRS	84.0	65.53 µg/mL	[189]
*L. plantarum*	Flask	Skim-milk-based media	-	910 µg/mL	[195]
*L. plantarum*	Flask	Goats-milk-based media	37.7	-	[200]
*L. rhamnosus*	Flask	Skim-milk-based media	-	700 µg/mL	[195]
*L. rhamnosus*	Flask	MRS	52.4	2130 µg/mL	[189]
*L. sakei*	Flask	Skim-milk-based media	-	1220 µg/mL	[195]
*Lc. lactis*	Flask	Skim-milk-based media	-	220 µg/mL	[195]
*E. durans*	Flask	Skim-milk-based media	-	450 µg/mL	[195]
*E. faecium*	Flask	MRS	55.4	70.5 µg/mL	[189]
*P. acidilactici*	Flask	MRS	84.7	19.78 µg/mL	[189]
*P. pentosaceus*	Flask	MRS	72.9	2070 µg/mL	[189]
*P. pentosaceus*	Flask	Skim-milk-based media	-	780 µg/mL	[195]
*S. thermophilus*	Flask	Skim-milk-based media	-	820 µg/mL	[195]
*L. delbrueckii* subsp. *bulgaricus* *L. reuteri* *Lc. lactic* supsp. *lactis*	Flask	Skim-milk-based media		610 µg/mL	[202]
*S. thermophilus*	Flask	Goats-milk-based media	40	-	[200]
*S. thermophilus*, *L.casei*, *L. helveticus*	Flask	Goats-milk-based media	82	-	[200]
*S. thermophilus*, *L. casei*, *L. plantarum*	Flask	Goats-milk-based media	43.3	-	[200]

**Table 8:** Summary of antioxidant peptide-producing LAB and their reported activity (DPPH: 1, 1-diphenyl-2-picrylhydrazyl; ABTS: 2,2′-Azino-bis-(3-ethylbenzothiazoline-6-sulfonic acid), diammonium salt; HFRSR: hydroxyl free radical scavenging rate).

Microorganism(s)	Fermentation	Media	DPPH (mg/mL) or %	ABTS (mg/mL)	HFRSR (%)	Antimutagenic Activity (% Inhibition)	Ref.
*S. thermophilus* *L. bulgaricus*	Cups	Skim-milk-based media	2.23 mg/mL	2.43 mg/ml	-	15.87	[214]
*S. thermophilus* *L. bulgaricus* *L. acidophilus*	Cups	Skim-milk-based media	2.05 mg/mL	2.28 mg/ml	-	18.35	[214]
*S. thermophilus* *L. bulgaricus* *L. casei*	Cups	Skim-milk-based media	1.83 mg/mL	1.91 mg/ml	-	18.83	[214]
*S. thermophilus* *L. bulgaricus* *L. paracasei*	Cups	Skim-milk-based media	1.82 mg/mL	1.98 mg/ml	-	18.48	[214]
*S. thermophilus* *L. bulgaricus* *L. acidophilus* *L. casei*	Cups	Skim-milk-based media	1.8 mg/mL	1.73 mg/ml	-	20.25	[214]
	Cups	Skim-milk-based media	1.77 mg/mL	1.8 mg/ml	-	23.06	[214]
*L. casei*	Flask	Goats-milk-based media	63.48%	-	88.01	-	[215]
*L. mesenteroides* subsp. *cremoris*	Flask	Skim-milk-based media	-	0.7 nmol^−1^/mmol^−1^	-	-	[216]
*L. lactis* subsp. *lactis*	Flask	Skim-milk-based media		0.15 nmol^−1^/mmol^−1^	-	-	[216]
*L. acidophilus*	Flask	Skim-milk-based media	-	0.6 nmol^−1^/mmol^−1^			[216]
*L. jensenii*	Flask	Skim-milk-based media	-	0.6 nmol^−1^/mmol^−1^	-	-	[216]
*L. helveticus*	Flask	Skim-milk-based media	-	0.4 nmol^−1^/mmol^−1^	-	-	[216]

**Table 9:** Summary of GABA-producing strains and yields (MRS: De Man, Rogosa, and Sharpe, MSG: monosodium glutamate, PLP: pyridoxal-5′-phosphate).

Microorganism (s)	Fermentation	Media	Yield	Ref.
*L. acidophilus*	Flask	Goats-milk-based media	1.92 mg/kg	[200]
*L. brevis*	Packed bed reactor	Defined media	55 mM	[229]
*L. brevis*	Batch	Modified MRS	44.4 mg/mL	[230]
*L. brevis*	Fed-batch	Defined media	1005.81 mM	[231]
*L. brevis*	Fed-batch	Complex	526.33 mmol/L	[232]
*L. brevis*	Flask	MRS + MSG	255 mM	[233]
*L. brevis*	Batch	Defined media	205.8 g/L	[234]
*L. brevis*	Batch	Defined media	62.5 g/L	[235]
*L. brevis*	Flask	Supplemented MRS	265 mM	[236]
*L. brevis*	Entrapped cell	Defined media + MSG,	223 mM	[237]
*L. brevis*	Flask	Whey-permeate-based media + MSG	553.5 mg/L	[238]
*L. brevis*	Flask	MRS + MSG	2.5 g/L	[239]
*L. brevis* *L. sakei*	Flask	Skim-milk-based media + MSG	22.41 mM	[240]
L. buchneri	Flask	Modified MRS	251 mM	[241]
*L. buchneri*	Flask	MRS + MSG	4.4 g/L	[242]
*L. casei*	Flask	Skim-milk-based media	677.35 mg/kg	[199]
*L. casei*	Flask	Goats-milk-based media	1.65 mg/kg	[200]
*L. delbrueckii* subsp. *bulgaricus*	Flask	Skim-milk-based media	9 mg/kg	[243]
*L. helveticus*	Flask	Skim-milk-based media	165.11 mg/L	[201]
*L. helveticus*	Flask	Goats-milk-based media	0.06 mg/kg	[200]
*L. paracasei*	Flask	Modified MRS	302 mM	[244]
*L. paracasei*	Flask	Skim-milk-based media	20 mg/kg	[243]
*L. paracasei* and *L. plantarum*	Flask	Skim-milk-based media	6.7 mg/100 mL	[245]
*L. plantarum*	Flask	Skim milk + MSG	629 mg/mL	[246]
*L. plantarum*	Flask	Defined media + MSG	19.8 g/L	[247]
*L. plantarum*	Flask	MRS	4.156 mg/L	[248]
*L. plantarum*	Flask	MRS + MSG	821.24 mg/L	[249]
*L. plantarum*	Flask	Skim-milk-based media + MSG	314 mg/100 g	[250]
*L. plantarum*	Flask	MRS + MSG	7.15 mM	[251]
*L. plantarum*	Flask	Grape must	4.85 mM	[252]
*L. plantarum*	Flask	MRS + MSG	201.78 mg/L	[253]
*L. plantarum*	Flask	Skim-milk-based media	6 mg/kg	[243]
*L. plantarum*	Flask	Skim milk + yeast extract	77.4 mg/kg	[195]
*L. plantarum*	Flask	Goats milk	12.84 mg/kg	[200]
*L. plantarum* and *L. sakei*	Flask	Whey-based media	365.6 mg/100 mL	[254]
*L. rhamnosus*	Flask	Complex media + MSG + PLP	187 mM	[255]
*L. sakei*	Batch	MRS + MSG	265.3 mM	[256]
*L. sakei*	Batch	MRS + MSG	217 mM	[256]
*S. salivarius* subsp. *thermophilus*	Flask	Complex media	7984.75 mg/L	[257]
*S. thermophilus*	Flask	Skim-milk-based media + MSG	2.2 mg/mL	[258]
*S. thermophilus*	Flask	Goats-milk-based media	1.59 mg/kg	[200]
*S. thermophilus* *L. brevis*	Flask	Skim milk + MSG	314.97 mg/kg	[259]
*S. thermophilus*, *L. casei*, *L. helveticus*	Flask	Goats-milk-based media	5.79 mg/kg	[200]
*S. thermophilus*, *L. casei*, *L. plantarum*	Flask	Goats-milk-based media	30.86 mg/kg	[200]

## Data Availability

Not applicable.
